# A dated phylogeny and collection records reveal repeated biome shifts in the African genus *Coccinia *(Cucurbitaceae)

**DOI:** 10.1186/1471-2148-11-28

**Published:** 2011-01-26

**Authors:** Norbert Holstein, Susanne S Renner

**Affiliations:** 1 Systematic Botany and Mycology, University of Munich (LMU), Menzinger Strasse 67, Munich, Germany

## Abstract

**Background:**

Conservatism in climatic tolerance may limit geographic range expansion and should enhance the effects of habitat fragmentation on population subdivision. Here we study the effects of historical climate change, and the associated habitat fragmentation, on diversification in the mostly sub-Saharan cucurbit genus *Coccinia*, which has 27 species in a broad range of biota from semi-arid habitats to mist forests. Species limits were inferred from morphology, and nuclear and plastid DNA sequence data, using multiple individuals for the widespread species. Climatic tolerances were assessed from the occurrences of 1189 geo-referenced collections and WorldClim variables.

**Results:**

Nuclear and plastid gene trees included 35 or 65 accessions, representing up to 25 species. The data revealed four species groups, one in southern Africa, one in Central and West African rain forest, one widespread but absent from Central and West African rain forest, and one that occurs from East Africa to southern Africa. A few individuals are differently placed in the plastid and nuclear (*LFY*) trees or contain two ITS sequence types, indicating hybridization. A molecular clock suggests that the diversification of *Coccinia *began about 6.9 Ma ago, with most of the extant species diversity dating to the Pliocene. Ancestral biome reconstruction reveals six switches between semi-arid habitats, woodland, and forest, and members of several species pairs differ significantly in their tolerance of different precipitation regimes.

**Conclusions:**

The most surprising findings of this study are the frequent biome shifts (in a relatively small clade) over just 6 - 7 million years and the limited diversification during and since the Pleistocene. Pleistocene climate oscillations may have been too rapid or too shallow for full reproductive barriers to develop among fragmented populations of *Coccinia, *which would explain the apparently still ongoing hybridization between certain species. Steeper ecological gradients in East Africa and South Africa appear to have resulted in more advanced allopatric speciation there.

## Background

Clades will typically retain their ecological characteristics, at least over moderate periods of evolutionary time [1,2], and where inherited climatic tolerances are narrow, this will limit species' geographic range expansion. As long as the inherited component of ecological preference is strong, species evolving in allopatry should initially have similar habitat requirements, and ecological differences between them should accumulate gradually [3]. These arguments set up expectations about how climate niches and species ranges in groups of related species should correlate with each other. Phylogeographic analyses of several African plant clades have found strong signal of Neogene habitat fragmentation and opportunity for allopatric speciation [4-8], but provided no quantitative data on ecological requirements of the species involved. Davis et al. [9] in their study of 11 species of the Malpighiaceae genus *Acridocarpus *showed that aridification in Eastern Africa apparently was accompanied by a small radiation, possibly involving niche shifts, but did not have details on species' drought tolerances. For a clade of tropical African Annonaceae, Couvreur et al. [10] inferred divergence events between East and West African rainforest species during the Pliocene and Miocen, but provided no data on niche shifts. A likely reason for the comparative neglect of tropical African plant groups in eco-evolutionary studies is that ranges are poorly known because the underlying occurrence data are too incomplete [11,12]. Related problems are a lack of monographic studies, poorly understood species boundaries, and few species-level phylogenies, the precondition for identifying sister species.

While African plant clades are thus underrepresented in eco-phylogenetic studies, the immense interest in primate evolution in Africa has resulted in a wealth of data on vegetation and climate history [13-15]. During the Middle Miocene, starting from about 16 Ma onwards, the African continent underwent gradual cooling and uplift in the east and south, leading to an expansion of woodlands and savannas, and reducing the ranges of lowland rain forest species [15-17]. By the Upper Miocene, 7 Ma ago, rifting and volcanism blocked precipitation, amplifying the overall aridification in East Africa [18,19]. The early Pliocene brought slightly warmer climates until c. 3.2 Ma [20], when the African tropics began experiencing dramatic climate changes that lasted throughout the Pleistocene and Holocene [21-24]. During the driest and coolest periods of the Pleistocene (2.6 Ma - 12,000 years ago), rain forests may have been restricted to refugia from which they re-expanded during more favorable periods [25-28]. The Quaternary climate oscillations affected all of equatorial Africa [29], with the most recent catastrophic destruction of rain forest occurring 2500 years ago [30].

Here we investigate clade diversification and changes in species' precipitation niches in the African cucurbit genus *Coccinia*. *Coccinia *comprises 27 species (all of them dioecious) and is almost confined to sub-Saharan Africa where it diversified into numerous habitat types. The only species that "escaped" from sub-Saharan Africa is *C. grandis*, which spread to the highlands of the Arabian Peninsula and tropical Asia, and is now an invasive weed on the Pacific Islands and in the Neotropics [31]. Pollination of *Coccinia *is by bees [[32]; NH, personal observation in Tanzania, August 2009], including honeybees. The numerous habitat types occupied by its 27 species make *Coccinia *a suiTable system in which to study niche evolution among close relatives. The niche parameters we focus on are annual precipitation and number of arid months, with species' tolerances being inferred from the occurrences of 1189 geo-referenced collections. Likely past changes in species' ecological preferences were inferred from a time-calibrated molecular tree including all but two of the species. We expected that close relatives would have similar climatic niche envelopes (e.g., drought tolerances), although clearly there had to have been at least two shifts since different *Coccinia *species occur in semi-arid habitats, woodland, and forest, vegetation types with contrasting precipitation regimes.

## Results

### Phylogenetic Reconstruction and Divergence Time Estimates

**Figure 1:**
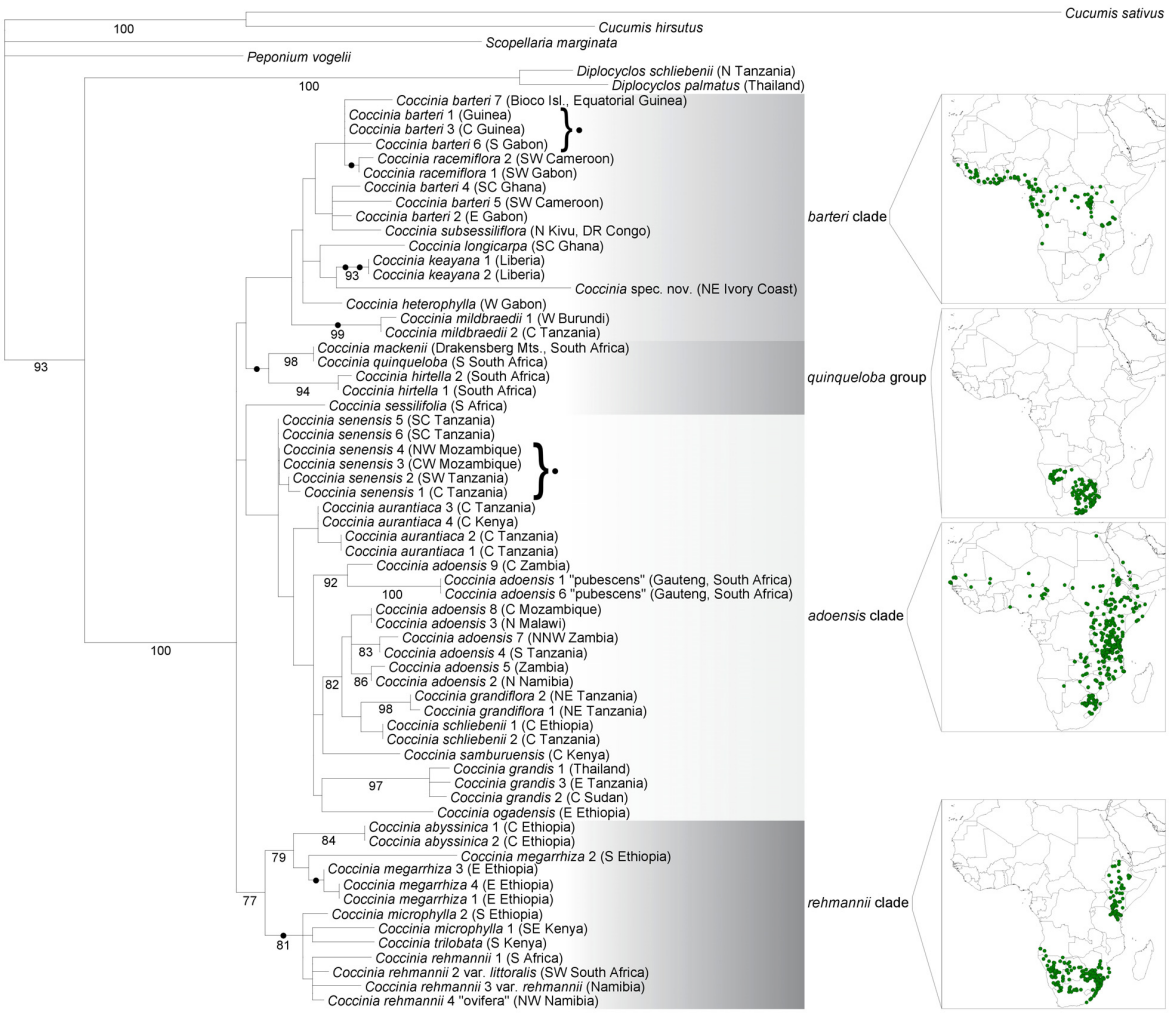
**Phylogeny for *Coccinia *based on plastid DNA sequence data**. Phylogenetic relationships in *Coccinia *based on 4,551 nucleotides of concatenated plastid DNA sequences obtained for 69 accessions and analyzed under maximum likelihood (ML) and the GTR + Γ model. Numbers below branches refer to ML bootstrap support > 75%. The dots at nodes and behind the two brackets refer to uniquely shared indels (*Methods*). The naming of the clades follows that in the nuclear tree (Figure 2). A single accession of *C. sessilifolia *in the nuclear tree groups with the *C. quinqueloba *clade, but the plastid data do not contain sufficient signal to resolve the placement of this species.

The concatenated plastid DNA alignment comprised 4551 nucleotides from 65 accessions representing 25 of the 27 species of *Coccinia*. Table 1 lists all DNA sources with their geographic origin, species name and author, and GenBank accession numbers. A maximum likelihood tree (Figure 1) obtained from the plastid data (TreeBASE accession 10846) shows four major groups: A *quinqueloba *group that occurs in southern Africa, a *barteri *clade that mostly occurs in Central and West African rain forest, an *adoensis *clade that is widespread, but absent from Central and West African rain forest, and a *rehmannii *clade that occurs from Ethiopia via East Africa to southern Africa.

**Table 1:** Voucher information and GenBank accession numbers

Species	No.	Voucher	Location	*mat*K	*ndh*F- *rpl*32R IS	*rpl*20- *rps*12 IS	*trn*L intron	*trn*L- *trn*F IS	*trn*S- *trn*G IS	*LFY *2^nd ^intron	ITS
*C. abyssinica *(Lam.) Cogn.	1	*E. Westphal & J. M. C. Westphal-Stevels *1552 (WAG)	Ethiopia, Oromia Region	HQ608224		HQ608311			HQ608429		
*C. abyssinica *(Lam.) Cogn.	2	*E. Westphal & J. M. C. Westphal-Stevels *1951 (WAG)	Ethiopia, Oromia Region			HQ608312	HQ608385	HQ608368	HQ608430		
*C. adoensis *(Hochst. ex A. Rich.) Cogn.	1	*L. E. Davidson *3781 (M)	South Africa, Gauteng	HQ608226	HQ608274	HQ608314	HQ608396	HQ608396	HQ608432		HQ608195
*C. adoensis *(Hochst. ex A. Rich.) Cogn.	2	*R. Story *6283 (M)	Namibia, Otjozondjupa	HQ608227	HQ608275	HQ608316	HQ608397	HQ608397	HQ608434	HQ608160	HQ608196 - 8
*C. adoensis *(Hochst. ex A. Rich.) Cogn.	3	*J. Pawek *6124 (MO)	Malawi, Northern Region	HQ608225		HQ608315		HQ608369	HQ608433		
*C. adoensis *(Hochst. ex A. Rich.) Cogn.	4	*R. E. Gereau & C. J. Kayombo *3582 (MO)	Tanzania, Iringa	HQ608231	HQ608273	HQ608313			HQ608431		
*C. adoensis *(Hochst. ex A. Rich.) Cogn.	5	*E. A. Robinson *2944 (M)	Zambia, Southern Prov.	HQ608228		HQ608318	HQ608398	HQ608398	HQ608436		HQ608199 - 201
*C. adoensis *(Hochst. ex A. Rich.) Cogn.	6	*H. Merxmüller *282 (M)	South Africa, Gauteng	HQ608229		HQ608319		HQ608370	HQ608437		
*C. adoensis *(Hochst. ex A. Rich.) Cogn.	7	*M. Sanane *375 (M)	Zambia, Northern Prov.	HQ608230		HQ608320	HQ608399	HQ608399	HQ608438		
*C. adoensis *(Hochst. ex A. Rich.) Cogn.	8	*A. R. Torre *5337 (M)	Mozambique, Zambezia			HQ608321		HQ608371	HQ608439		
*C. adoensis *(Hochst. ex A. Rich.) Cogn.	9	*D. K. Harder & M. G. Bingham *2584 (MO)	Zambia, Lusaka Prov.	HQ608268	HQ608299	HQ608364			HQ608492	HQ608191	HQ608221
*C. aurantiaca *C. Jeffrey	1	*M. Richards *20987 (BR)	Tanzania, Iringa	HQ608235		HQ625507	HQ608401	HQ608401	HQ608443		
*C. aurantiaca *C. Jeffrey	2	*P. J. Greenway & Kanuri *14811 (M)	Tanzania, Iringa				HQ608402	HQ608402	HQ608444	HQ608161	HQ608202
*C. aurantiaca *C. Jeffrey	3	*N. Holstein *et al. 86 (M)	Tanzania, Dodoma	HQ608236	HQ608276	HQ608325	HQ608403	HQ608403	HQ608445	HQ608162	
*C. aurantiaca *C. Jeffrey	4	*S. A. Robertson *1925 (MO)	Kenya, Eastern Prov.	HQ608232		HQ608322	HQ608400	HQ608400	HQ608440		
*C. barteri *(Hook. f.) Keay	1	*E. Achigan-Dako *07 NIA 899 (GAT)	Guinea, Nzérékoré Region	HQ608237		HQ608330	HQ608404	HQ608404	HQ608450		HQ608203
*C. barteri *(Hook. f.) Keay	2	*J. J. Wieringa *6387 (WAG)	Gabon, Haut-Ogooué	HQ608239	HQ608277	HQ608326	HQ608405	HQ608405	HQ608446	HQ608163	HQ608204
*C. barteri *(Hook. f.) Keay	3	*E. Achigan-Dako *06 NIA 294 (GAT)	Guinea, Mamou Region			HQ608331	HQ608389	HQ608376	HQ608451		
*C. barteri *(Hook. f.) Keay	4	*E. Achigan-Dako *07 NIA 809 (GAT)	Ghana, Eastern Region	HQ608240		HQ608327	HQ608387	HQ608374	HQ608447	HQ608164	
*C. barteri *(Hook. f.) Keay	5	*W. J. J. O. de Wilde *et al. 3736 (MO)	Cameroon, Central Region	HQ608241		HQ608328	HQ608388	HQ608375	HQ608448		
*C. barteri *(Hook. f.) Keay	6	*M. A. van Bergen *490 (WAG)	Gabon, Ogooué-Maritime	HQ608242	HQ608278	HQ608329	HQ608406	HQ608406	HQ608449	HQ608165	
*C. barteri *(Hook. f.) Keay	7	*F. J. Fernández-Casas *12077 (MO)	Equatorial Guinea, Bioco Island	HQ608238	HQ608279	HQ608332	HQ608390	HQ608377	HQ608453		
*C. grandiflora *Cogn.	1	*H. Schäfer *05/302 (M)	Tanzania, Tanga	HQ608243	HQ608280	HQ608333	HQ608407	HQ608407	HQ608454	HQ608166	HQ608205
*C. grandiflora *Cogn.	2	*N. Holstein *et al. 98 (M)	Tanzania, Tanga	HQ608244	HQ608281	HQ608334	HQ608408	HQ608408	HQ608455	HQ608167	
*C. grandis *(L.) Voigt	1	*W. J. J. O. de Wilde & B. E. E. Duyfjes *22270 (L)	Thailand, Bangkok	DQ536651	HQ608282	DQ536537	DQ536762	DQ536762	HQ608456	HQ608168	HQ608207
*C. grandis *(L.) Voigt	2	*R. Müller *s.n., Aug. 1999 (MSB)	Sudan, Sannar Prov.			HQ608335	HQ608409	HQ608409	HQ608457	HQ608169	
*C. grandis *(L.) Voigt	3	*H. Schäfer *05/258 (M)	Tanzania, Pwani	HQ608245	HQ608283	HQ608336	HQ608410	HQ608410	HQ608458	HQ608170	HQ608206
*C. heterophylla *(Hook. f.) Holstein		*C. C. H. Jongkind *5905 (WAG)	Gabon, Estuaire	HQ608246		HQ608337	HQ608411	HQ608411	HQ608459	HQ608171	
*C. hirtella *Cogn.	1	*N. Holstein *29 (M)	J.-L. Gatard, France, wild source unknown	HQ608247	HQ608284	HQ608339	HQ608412	HQ608412	HQ608461	HQ608172	
*C. hirtella *Cogn.	2	*S. S. Renner & A. Kocyan *2447 (M)	J.-L. Gatard, France, wild source unknown	HQ608248		HQ608338	HQ608413	HQ608413	HQ608460		
*C. keayana *R. Fernandes	1	*F. C. Straub *140 (BR)	Liberia						HQ608462		
*C. keayana *R. Fernandes	2	*C. C. H. Jongkind *et al. 6542 (WAG)	Liberia, Grand Gedeh	HQ608249	HQ608285	HQ608340		HQ608378	HQ608463	HQ608173	HQ608211
*C. longicarpa *Jongkind		*C. C. H. Jongkind *3970 (WAG)	Ghana, Ashanti Region	HQ608250	HQ608286	HQ608341	HQ608414	HQ608414	HQ608464	HQ608174	HQ608212
*C. mackenii *Naudin ex C. Huber		*R. G. Strey *3762 (M)	South Africa, Mpumalanga	HQ608251		HQ608343	HQ608415	HQ608415	HQ608465		
*C. megarrhiza *C. Jeffrey	1	*J. J. F. E. de Wilde *6501 (WAG)	Ethiopia, Oromia Region			HQ608344	HQ608417	HQ608417	HQ608466		
*C. megarrhiza *C. Jeffrey	2	*I. Friis *et al. 2664 (MO)	Ethiopia, Oromia Region	HQ608252	HQ608287	HQ608347	HQ608416	HQ608416	HQ608469	HQ608176	
*C. megarrhiza *C. Jeffrey	3	*P. C. M. Jansen *3471 (WAG)	Ethiopia, Oromia Region			HQ608345			HQ608467		
*C. megarrhiza *C. Jeffrey	4	*J. J. F. E. de Wilde *4793 (WAG)	Ethiopia, Oromia Region	HQ608253		HQ608346			HQ608468	HQ608175	
*C. microphylla *Gilg	1	*R. B. Drummond & J. H. Hemsley *4087 (B)	Kenya, Coast Province	HQ608254		HQ608348			HQ608470	HQ608177	
*C. microphylla *Gilg	2	*J. J. F. E. de Wilde & M. G. Gilbert *346 (UPS)	Ethiopia, Somali Regional State	HQ608255		HQ608349	HQ608418	HQ608418	HQ608471	HQ608178	HQ608213
*C. mildbraedii *Gilg ex Harms	1	*M. Reekmans *7399 (BR)	Burundi, Muramvya Prov.	HQ608256		HQ608350			HQ608472		
*C. mildbraedii *Gilg ex Harms	2	*N. Holstein *et al. 76 (M)	Tanzania, Morogoro	HQ608257	HQ608288	HQ608351	HQ608419	HQ608419	HQ608473	HQ608179	
*C. ogadensis *Thulin		*M. Thulin *et al. 11183 (UPS)	Ethiopia, Somali Regional State	HQ608258	HQ608289	HQ608352			HQ608474		HQ608214 - 6
*C. quinqueloba *(Thunb.) Cogn.		*R. D. A. Bayliss *8470 (M)	South Africa, Eastern Cape	HQ608259	HQ608290	HQ608353	HQ608420	HQ608420	HQ608475	HQ608180	
*C. racemiflora *Kéraudren	1	*I. van Nek *536 (WAG)	Gabon, Ogooué-Maritime			HQ608355	HQ608421	HQ608421	HQ608477	HQ608182	HQ608217
*C. racemiflora *Kéraudren	2	*J. J. Bos *6590 (WAG)	Cameroon, South Prov.	HQ608260		HQ608354	HQ608391	HQ608379	HQ608476	HQ608181	
*C. rehmannii *Cogn.	1	*S. S. Renner & A. Kocyan *2749 (M)	southern Africa, no detailed information	DQ536652	HQ608292	HQ625508	DQ536799	DQ536799	HQ608479	HQ608184	HQ608218
*C. rehmannii *Cogn. var. *littoralis *A. Meeuse	2	*L. E. Codd *9620 (M)	South Africa, KwaZulu-Natal	HQ608261		HQ625509	HQ608422	HQ608422	HQ608480		
*C. rehmannii *Cogn. var. *rehmannii*	3	*G. Woortman *217 (M)	Namibia, Otjozondjupa	HQ608262		HQ625510	HQ608392	HQ608380	HQ608481	HQ608185	
*C. rehmannii *Cogn.*"ovifera"*	4	*B. de Winter & O. A. Leistner *5598 (M)	Namibia, Kunene	HQ608263	HQ608291	HQ608356	HQ608423	HQ608423	HQ608478	HQ608183	
*C. samburuensis *Holstein		*R. B. & A. J. Faden *74/948 (WAG)	Kenya, Rift Valley Prov.	HQ608264	HQ608293	HQ608357	HQ608393	HQ608381	HQ608482	HQ608186	
*C. schliebenii *Harms	1	*E. Westphal & J. M. C. Westphal-Stevels *5539 (WAG)	Ethiopia, Oromia Region		HQ608294	HQ608358			HQ608483		
*C. schliebenii *Harms	2	*G. S. Laizer *et al. 1449 (MO)	Tanzania, Morogoro	HQ608265		HQ608359		HQ608382	HQ608484	HQ608187	
*C. senensis *(Klotzsch) Cogn.	1	*N. Holstein *et al. 66 (M)	Tanzania, Morogoro	HQ608266	HQ608295	HQ608360	HQ608424	HQ608424	HQ608485	HQ608188	
*C. senensis *(Klotzsch) Cogn.	2	*K. Vollesen *MRC4316 (WAG)	Tanzania, Lindi	HQ608267	HQ608296	HQ608362	HQ608425	HQ608425	HQ608487	HQ608189	HQ608219
*C. senensis *(Klotzsch) Cogn.	3	*A. R. Torre *et al. 18788 (MO)	Mozambique, Tete			HQ608361			HQ608486		
*C. senensis *(Klotzsch) Cogn.	4	*E. M. C. Groenendijk *et al. 1031 (WAG)	Mozambique, Nampula			HQ625511			HQ608489		
*C. senensis *(Klotzsch) Cogn.	5	*J. Lovett *1597 (MO)	Tanzania, Iringa	HQ608233		HQ608323	HQ608386	HQ608372	HQ608441		
*C. senensis *(Klotzsch) Cogn	6	*C. F. Paget-Wilkes *72 (MO)	Tanzania, Iringa	HQ608234		HQ608324		HQ608373	HQ608442		
*C. sessilifolia *(Sond.) Cogn.		*S. S. Renner *et al. 2763 (M)	Plant grown at Mainz Bot. G. (MJG19-54430); wild source unknown	AY968446	HQ608297	DQ648163	AY968568	AY968385	HQ608490	HQ608190	HQ608220
*C. *spec. nov.		*C. Geerling & J. Bokdam *662 (MO)	Ivory Coast, Bouna area	HQ608269	HQ608298	HQ608363		HQ608383	HQ608491		
*C. subsessiliflora *Cogn.		*H. F. in de Witte *8288 (M)	DR Congo, Kivu	HQ608270		HQ608365	HQ608395	HQ608384	HQ608493		
*C. trilobata *(Cogn.) C. Jeffrey		*N. Holstein & P. Sebastian *9 (M)	J.-L. Gatard, France, coll. in Kenya	HQ608271	HQ608300	HQ608366	HQ608426	HQ608426	HQ608494		HQ608222
*Diplocyclos palmatus *(L.) C. Jeffrey		*J. Maxwell *s.n. 2 Sep. 2002	Thailand, Chiang Mai	DQ536671	HQ608301	DQ536625	DQ536769	DQ536769	HQ608495	HQ608192	
*Diplocyclos schliebenii *(Harms) C. Jeffrey		*H. J. Schlieben *4363 (M)	Tanzania, Kilimanjaro				HQ608427	HQ608427	HQ608496	HQ608193	HQ608223
*Cucumis hirsutus *Sond.		*N. B. Zimba *et al. 874 (MO)	Zambia	DQ536658		DQ536542	DQ536804	DQ536804	HM597074		
*Cucumis sativus *L.		Unknown	unknown	AJ970307	AJ970307	AJ970307	AJ970307	AJ970307	AJ970307		
*Peponium vogelii *(Hook. f.) Engl.		*S. S. Renner *2710 (M)	Tanzania, Tanga	HQ608272	HQ608302	HQ608367	HQ608428	HQ608428	HQ608497	HQ608194	
*Scopellaria marginata *(Bl.) W. de Wilde and Duyfjes		*A. Kocyan *AK178 (BKF)	Thailand	DQ536751		DQ536612	DQ536804	DQ536804			

**Figure 2:**
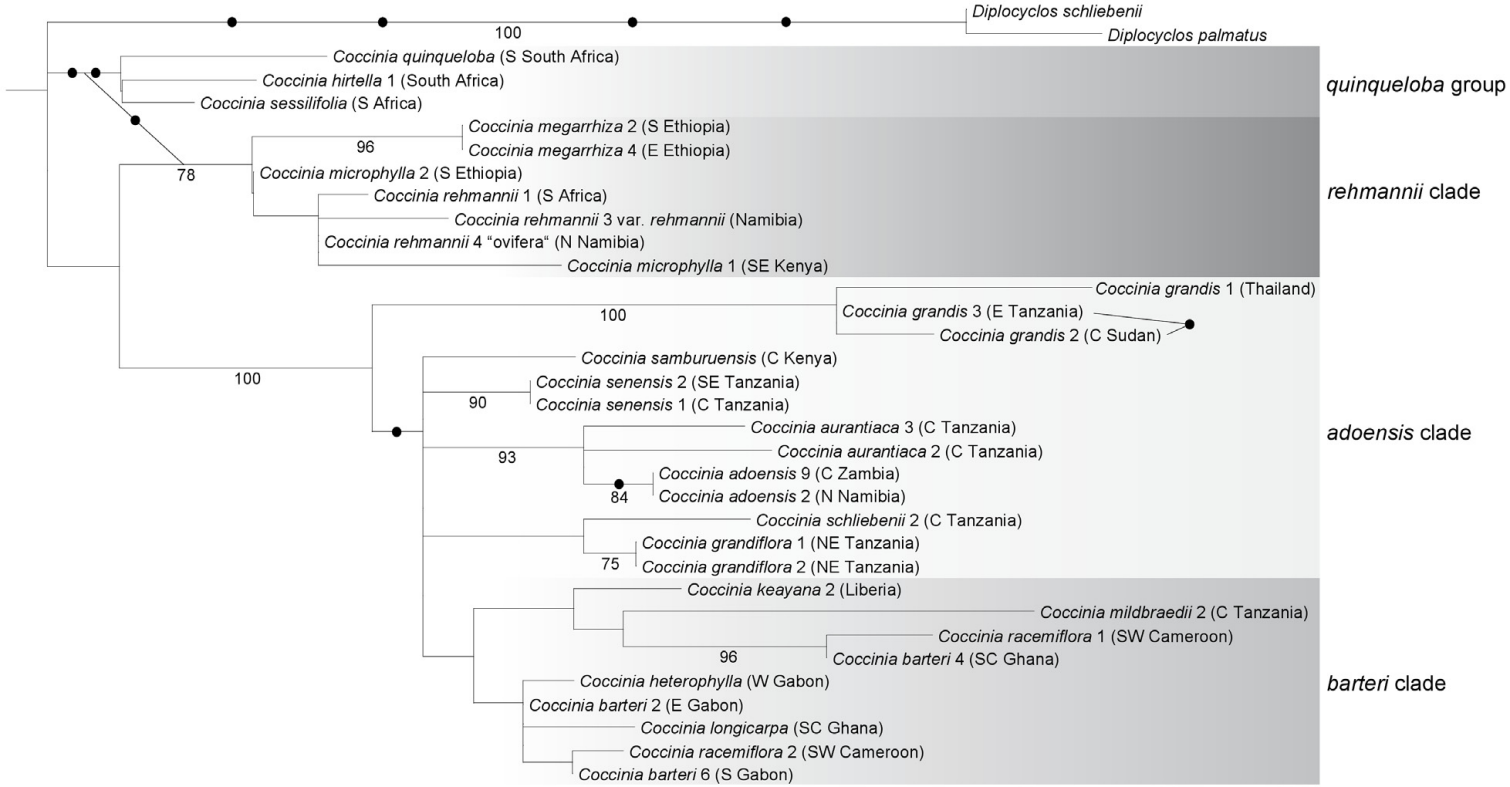
**Phylogeny for *Coccinia *based on nuclear data**. Phylogenetic relationships in *Coccinia *based on 463 nucleotides of the nuclear *LFY *2^nd ^intron, obtained for 35 accessions from 23 species analyzed under maximum likelihood (ML) and the GTR + Γ model. Numbers below branches refer to ML bootstrap support > 75%. The dots at nodes and behind the two brackets refer to uniquely shared indels. The tree was rooted on *Peponium vogelii *(not shown).

The nuclear *LFY *2^nd ^intron alignment (TreeBASE accession 10846) comprised 463 characters for 35 accessions, representing 20 species of *Coccinia *plus three outgroups. A maximum likelihood tree from these data (Figure 2) does not contradict the plastid tree topology except for a few accessions in the *C. adoensis *and *C. barteri *clades discussed below, and an accession of *C. sessilifolia*, which in the nuclear tree groups with the *quinqueloba *group, but in the plastid tree groups with the *adoensis *clade.

**Figure 3:**
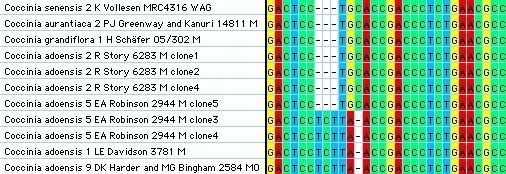
**Detail of the nuclear ITS alignment for *Coccinia***. Section of the aligned *Coccinia *nuclear internal transcribed spacer (ITS) sequences, indicating gene flow between individuals of *C. adoensis *with different plastid genotypes.

A tree from the nuclear ITS alignment is almost unresolved (data not shown), but ITS sequences helped pinpoint suspected hybridization (Figure 3; see the section on **Evidence for Hybridization**). For example, individuals of *C. adoensis *from different parts of the species' range have different ITS sequences.

**Figure 4:**
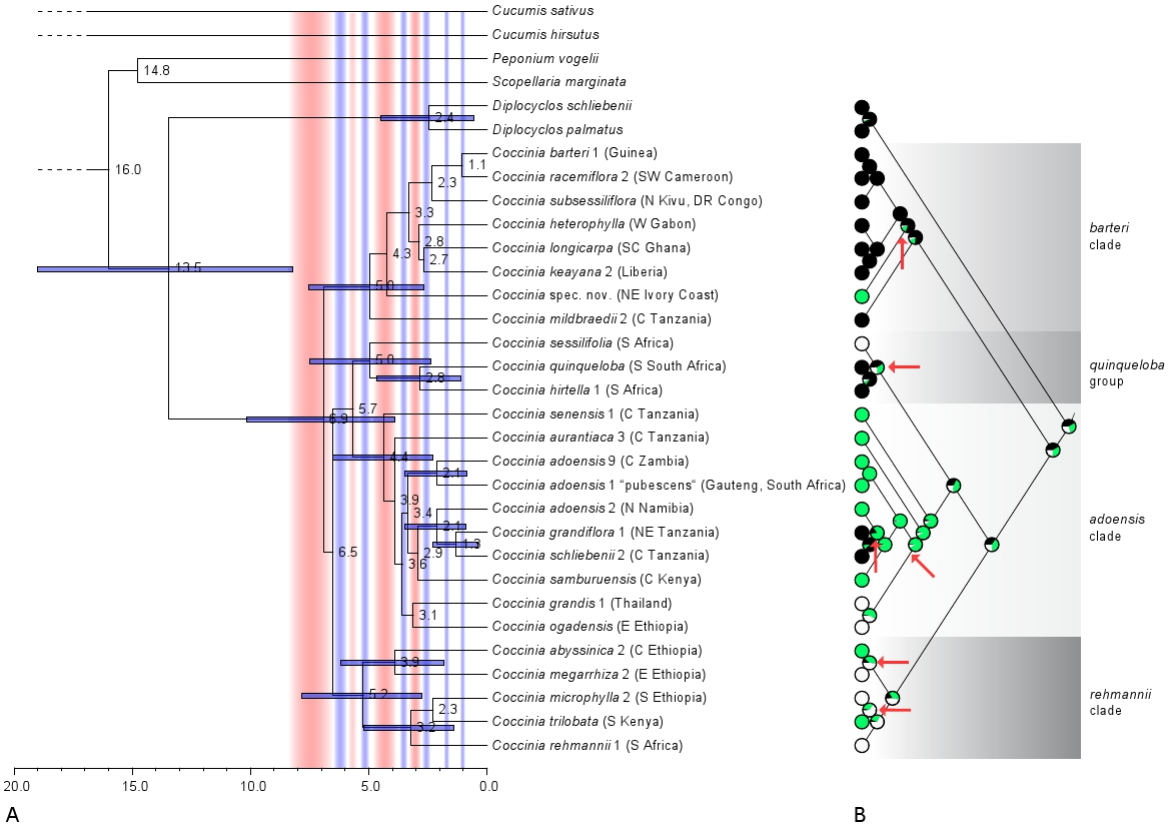
**Chronogram and ancestral character reconstruction in *Coccinia***. a. Chronogram for 24 *Coccinia *species (*C. mackenii *and *C. quinqueloba *had identical sequences, and the former was therefore removed; *Methods*) obtained under a Bayesian strict clock model. Clades are labeled as in Figure 1 and 2; blue bars around mean node ages represent the 95% highest posterior density credibility interval, and are given for all nodes with ≥ 0.98 posterior probability. Pale red backgrounds indicates warm (humid) climate, pale blue backgrounds, cold (arid) climates after [15]. b. Ancestral biome reconstruction on the Bayesian tree obtained from the plastid DNA data for *Coccinia*; Black = forest, green = woodland and semi-humid savannas, and white = semi-arid habitats. Red arrows indicate biome shifts.

A chronogram from a slightly reduced plastid DNA data set (Figure 4a) shows the inferred absolute ages (with 95% confidence intervals) for nodes with >0.98 posterior probability. The diversification of *Coccinia *apparently began 6.9 Ma ago (10.2 - 3.9 Ma, 95% highest posterior density [HPD]), with most of the extant species diversity dating to the Pliocene.

### Climatic Tolerances and Biome Preferences among Close Relatives

Differences in climatic tolerances for species in well-supported clades were quantified by pair-wise Mann-Whitney U tests, focusing on annual precipitation and number of arid months (Table 2a - c). After each species or unit (in the case of the three genotypes of *C. adoensis*) had been assigned to one of three habitat categories (semi-arid habitats, woodland, or forest; *see Methods*), maximum likelihood inference of habitat shifts on the phylogeny and the Mann-Whitney U tests revealed at least six habitat changes (marked by red arrows in Figure 4b), counting only changes in statistically supported sister species or clades. Differentiation of precipitation preferences within habitat category (e.g., in *C. quinqueloba *versus *C. mackenii*, Table 2a) was not counted as a biome shift.

**Table 2:** Pairwise Mann-Whitney U tests among species of supported clades in the *Coccinia *phylogeny

a. Pairwise Mann-Whitney U tests among species of the *Coccinia rehmannii *clade and the *C. quinqueloba *group										
	*abyssinica*	*megarrhiza*	*microphylla*	*trilobata*	*rehmannii*	*quinqueloba*	*mackenii*	*hirtella*	*sessilifolia*	

*abyssinica*	-	0.047*								
*megarrhiza*	< 0.001**	-								
*microphylla*			-	< 0.001**	0.971					
*trilobata*			< 0.001**	-	< 0.001**					
*rehmannii*			0.128	< 0.001**	-					
*quinqueloba*						-	0.004*	0.013*	< 0.001**	
*mackenii*						< 0.001**	-	0.206	< 0.001**	
*hirtella*						< 0.001**	0.003*	-	< 0.001**	
*sessilifolia*						< 0.001**	< 0.001**	< 0.001**	-	

**b. Pairwise Mann-Whitney U tests among species of the *Coccinia barteri *clade**										

	*barteri*	*racemiflora*	*subsessiliflora*	*longicarpa*	*keayana*	*heterophylla*	spec. nov.	*mildbraedii*		

*barteri*	-	0.336	0.001*	0.087	0.056	0.001*	0.006*	0.335		
*racemiflora*	0.009*	-	0.026*	0.094	0.091	0.445	0.095	0.601		
*subsessiliflora*	0.63	< 0.001**	-	0.027*	0.077	< 0.001**	0.017*	0.045*		
*longicarpa*	0.771	< 0.001**	0.251	-	0.746	< 0.001**	0.011*	0.94		
*keayana*	0.009*	0.968	0.002*	0.012*	-	< 0.001**	0.026*	0.871		
*heterophylla*	0.016*	0.042*	0.041*	0.018*	0.002*	-	0.029*	0.006*		
spec. nov.	0.093	0.095	0.017*	0.042*	0.013*	0.941	-	0.003*		
*mildbraedii*	0.004*	< 0.001**	0.036*	0.002*	< 0.001**	0.148	0.139	-		

**c. Pairwise Mann-Whitney U tests among species of the *Coccinia adoensis *clade**										

	*senensis*	*aurantiaca*	*adoensis *9	*adoensis "pubescens"*	*adoensis*	*grandiflora*	*schliebenii*	*samburuensis*	*ogadensis*	*grandis*

*senensis*	-	0.241	0.333	< 0.001**	0.549	< 0.001**	< 0.001**	0.333	< 0.001**	0.531
*aurantiaca*	0.001*	-	0.625	< 0.001**	0.1	< 0.001**	< 0.001**	0.961	< 0.001**	0.35
*adoensis *9	0.444	0.75	-	0.03*	0.281	0.043*	0.071	0.4	0.222	0.705
*adoensis "pubescens"*	< 0.001**	0.352	0.636	-	< 0.001**	0.001*	0.15	< 0.001**	< 0.001**	0.091
*adoensis*	0.098	0.01*	0.607	< 0.001**	-	< 0.001**	< 0.001**	0.24	< 0.001**	0.903
*grandiflora*	0.015*	< 0.001**	0.043*	< 0.001**	< 0.001**	-	0.138	< 0.001**	< 0.001**	< 0.001**
*schliebenii*	< 0.001**	< 0.001**	0.071	< 0.001**	< 0.001**	0.014*	-	0.002*	< 0.001**	0.061
*samburuensis*	0.002*	0.185	0.4	0.061	0.006*	< 0.001**	< 0.001**	-	0.004*	0.521
*ogadensis*	< 0.001**	< 0.001**	**0.222**	< 0.001**	< 0.001**	< 0.001**	< 0.001**	0.004*	-	0.002*
*grandis*	0.02*	0.542	0.914	0.219	0.03*	< 0.001**	< 0.001**	0.314	< 0.001**	-

Fields above the em dash (-) line are comparisons of the number of arid months, below are comparisons of the annual precipitation. One asterisk (*) indicates significance at the 5% level, two asterisks (**) indicate significance at the 0.1% level. Bold numbers indicate statistically significant differentiation between species (biome switch).

We next tested whether the number of pairs of species that have the same niche preferences differs from that obtained if species habitat distributions were drawn at random (proportion 0.635). Among the 27 nodes in the phylogeny, 6 involved habitat shifts (red arrows in Figure 4), which is significantly fewer than the expected number of 17 (*G *= 9.4, df = 1, *P *= 0.0021). Even when the four most basal nodes are deleted from the analysis owing to the ambiguity of their character states, the phylogeny still includes significantly fewer habitat shifts than expected at random (*G *= 6.5, df = 1, *P *= 0.011). Thus, occupation of one of our three habitat categories appears to be a statistically conservative trait in the sense that daughter lineages tend to retain habitat type more frequently than expected by chance, given that the random probabilities are estimated from the current distributions of species. The next sections briefly describe the geography and timing of the inferred six shifts between semi-arid habitats, woodland, and forest.

**Figure 5:**
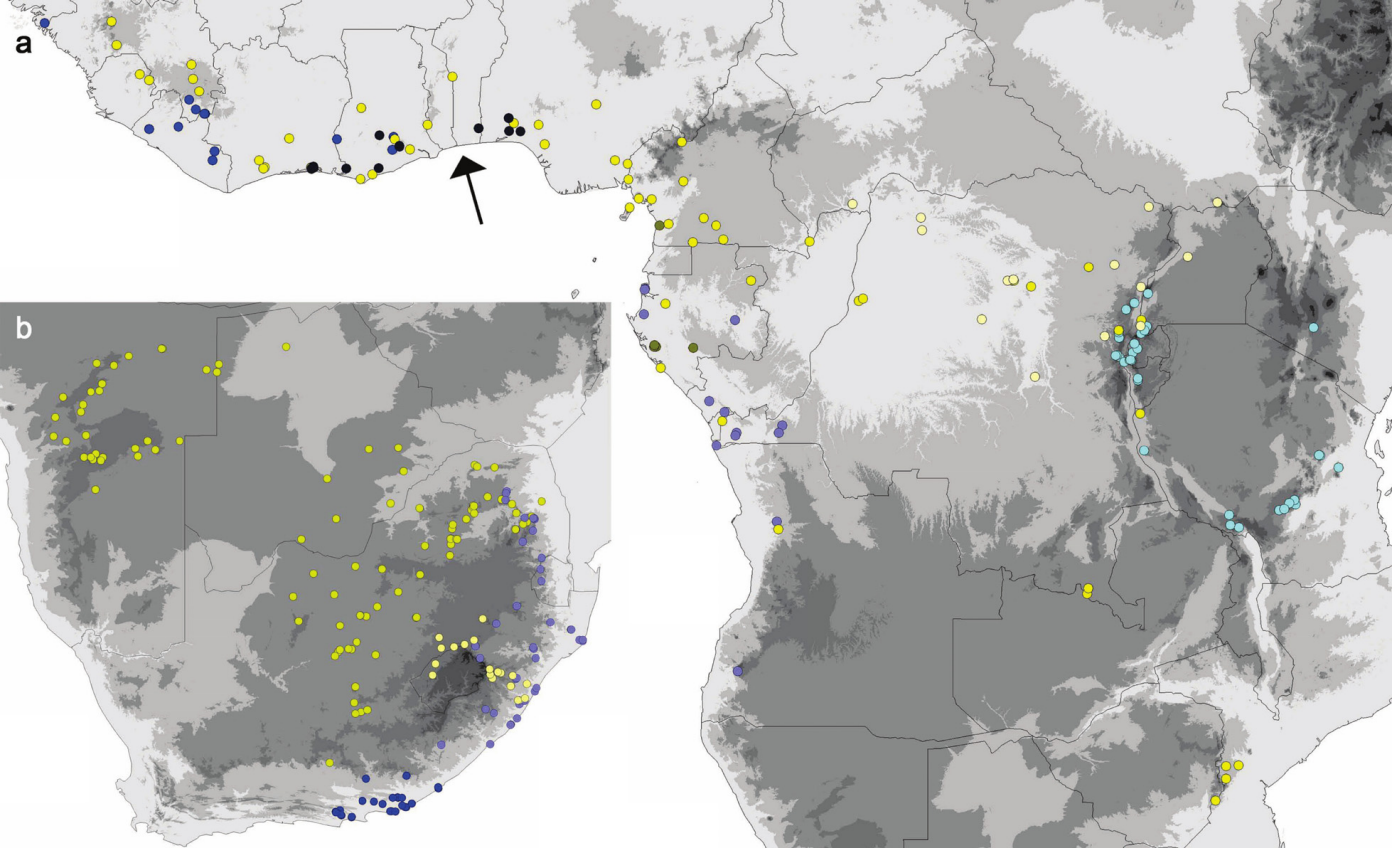
**Distribution of species in the *Coccinia barteri *clade and the *C. quinqueloba *clade**. a Distribution of species in the *Coccinia barteri *clade. Dark blue = *C. longicarpa*, deep blue = *C. keayana*, pale blue = *C. heterophylla*, light blue = *C. mildbraedii*, bright yellow = *C. barteri *morphs, dark yellow = *C. racemiflora*, pale yellow = *C. subsessiliflora*. The arrow marks the Dahomey Gap. b Distribution of species in the *quinqueloba *group. Dark blue = *C. quinqueloba*, pale blue = *C. mackenii*, bright yellow = *C. sessilifolia*, pale yellow = *C. hirtella*.

**Figure 6:**
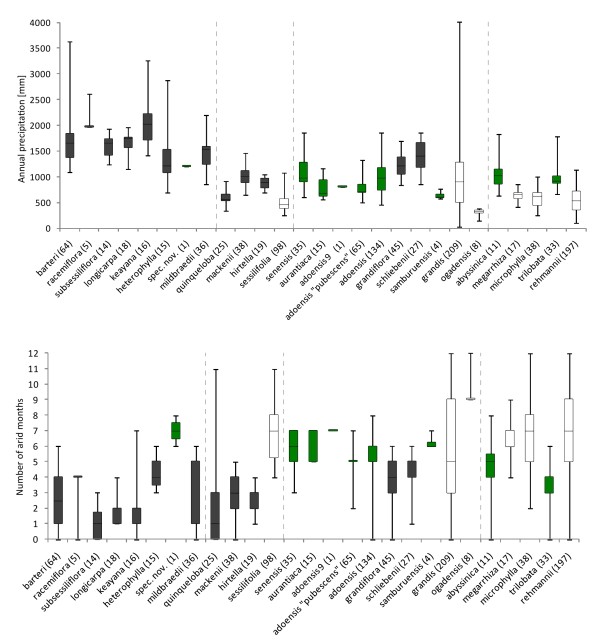
**Precipitation variation of species in the genus *Coccinia***. Box-and-whisker plots displaying the median, upper quartile, and lower quartile (box) and range of extremes (whiskers) of the precipitation tolerances (mean annual precipitation and number of arid months) of 1189 georeferenced herbarium collections. Numbers of collections for each species are given in the parentheses following the species names, and the species order follows the phylogeny in Figure 4. Dashed lines separate the clades/groups, and colors indicate the biomes: black = forest, green = woodland and humid open habitats, and white = semi-arid habitats.

The *Coccinia quinqueloba *group comprises four species and began diversifying c. 5 Ma (7.5 - 2.4 95% HPD) ago. Its divergence times and habitat preferences are shown in Figure 4, geographic ranges in Figure 5b, and precipitation tolerances in Figure 6. The species in this group occur in two habitat categories (forest and semi-arid habitat), and there was at least one niche shift in terms of the tolerated precipitation regime. The three forest species (of which *C. mackenii *and *C. quinqueloba *have identical sequences in 3503 nucleotides) diverged around 2.8 Ma (4.7 - 1.1 95% HPD) ago, during the Late Pliocene to Pleistocene.

The *Coccinia barteri *clade includes eight Central and West African species plus the East African (Tanzanian) *C. mildbraedii *(incl. *C. ulugurensis*); Figure 5a shows the species' geographic ranges (except for *Coccinia *spec. nov.; Table 1 provides the vouchers and code numbers for each sequenced plant) and Figure 6 their climatic tolerances. Diversification of this clade began 5 Ma (7.6 - 2.7 95% HPD; Figure 4a) ago, that is, at the beginning of the Early Pliocene warming. Most of the species occur in lowland rain forests or mountain forests at elevations up to 2900 m, although *C. barteri *and *C. heterophylla *also have been collected in humid semi-deciduous forests and clearings. *Coccinia *spec. nov. represents a biome shift from rain forest to semi-humid savanna (our woodland category). *Coccinia barteri *is morphologically diverse, and based on herbarium material, species boundaries in the *barteri *clade tend to be cryptic (Table 3).

**Table 3:** Key characters among forest species in the *Coccinia barteri *clade, illustrating the high level of morphological differentiation among close relatives (Figure 1 and 2)

Species / accession	Male raceme morphology	Bracts	Calyx teeth	Fruit shape	Other characters
*C. mildbraedii*	Condensed on long stalk	No	Upright, short, acute	Long cylindrical	Tendrils bifid
*C. keayana*	Lax, many-flowered	No	Erect-reflexed, long, narrow	Ovoid	Tendrils simple
*C. longicarpa*	Condensed	No	Erect-upright, broad	Long cylindrical	Tendrils simple (rarely bifid)
*C. heterophylla*	Condensed (rarely also lax)	Yes	Upright, long subulate	Ovoid	Tendrils bifid
*C. subsessiliflora*	Condensed, few-flowered	Yes	Upright, short, acute	Ovoid	Tendrils simple; leaves more deeply lobate than in other species
*C. racemiflora*	Lax, many-flowered	No	Erect-upright, slightly fleshy, short, narrow	Ovoid	Tendrils bifid
*C. barteri *(type morph)	Condensed, many-flowered	Yes	Upright, short, acute	Ovoid	Tendrils simple or bifid
*C. barteri *7-like	Condensed	Yes	Reflexed, fleshy, short	Ovoid	Tendrils simple or bifid
*C. barteri *2	Condensed, few-flowered	No	Erect-reflexed, short, subulate	Ovoid	Tendrils bifid
*C. barteri *4	Condensed, few-flowered	Yes	Upright, short, acute	?	Tendrils simple or bifid
*C. barteri *5	Condensed, few-flowered	No	Erect, short, acute	?	Tendrils simple
*C. barteri *6 (x*racemiflora*?)	Condensed, but pedicels rather long	No	Upright, short, acute	?	Tendrils bifid

**Figure 7:**
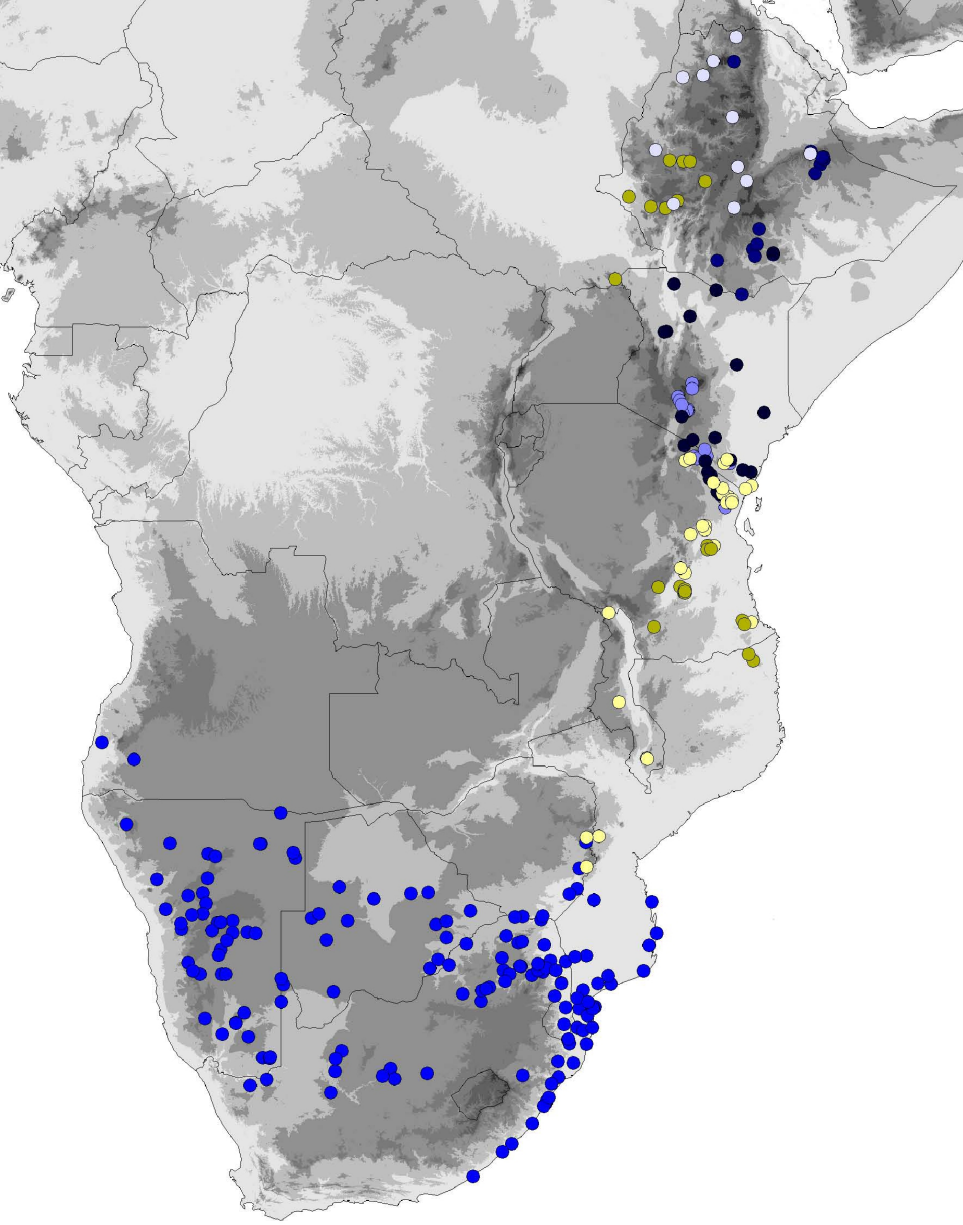
**Distribution of *C. grandiflora, C. schliebenii*, and species of the *Coccinia rehmannii *clade**. Bright blue (southern Africa) = *C. rehmannii*, pale blue = *C. trilobata*, blackish blue = *C. microphylla*, ice-blue = *C. abyssinica*, dark blue = *C. megarrhiza*. Bright yellow = *C. schliebenii*, pale yellow = *C. grandiflora*.

The *Coccinia adoensis *clade comprises nine species and includes at least two biome shifts (Figure 4b). The first involves the sister species *C. grandiflora *and *C. schliebenii*, which occur in (rain-) forests of East Africa (Figure 4b and 7), while their widespread relative *C. adoensis *occurs in mountain grasslands, deciduous woodlands, and rarely in moister bushlands (> 450 mm annual precipitation, < 7 months of aridity) from South Africa to Ethiopia and to Nigeria. The deeper split is dated to the Late Pleistocene (c. 2.1 Ma ago), while *C. grandiflora/C. schliebenii *separated from each other c. 1.3 Ma ago (Figure 4a). The second biome shift involves *C. ogadensis *and *C. grandis*, which are adapted to semi-arid conditions (Figure 4b).

The *Coccinia rehmannii *clade, which started diversifying during an arid period at the end of the Miocene 5.2 Ma (7.9 - 2.8 95% HPD) ago (Figure 4a), comprises five species (Figure 7: blue dots) and two biome switches (Figure 4b). The split between *C. abyssinica *and *C. megarrhiza *dates to c. 3.9 Ma ago, at the end of the warm and humid Early Pliocene, and that of *C. rehmannii *from *C. microphylla *and *C. trilobata *to c. 3.2 Ma, during the humid Late Pliocene. The climate tolerances of the five species are shown in Figure 6.

### Evidence for Hybridization

One of the incongruities between the nuclear and plastid DNA tree topologies concerns *C. racemiflora *from rain forests of west equatorial Africa. In the plastid tree (Figure 1), *C. racemiflora *1 and *C. racemiflora *2 group together and share a 490 bp deletion in *trn*S^GCU^-*trn*G^UCC ^intergenic spacer. Morphologically, these two plants appear to represent the same species. However, in the nuclear *LFY *tree (Figure 2), *C. racemiflora *1 groups with *C. barteri *4 while *C. racemiflora *2 groups with *C. barteri *6. The latter plant is morphologically intermediate between *C. barteri *and *C. racemiflora *and may present a hybrid.

Two other incongruities concern *C. adoensis *(compare accessions 1 to 9 in Figure 1-3). First, in the plastid tree (Figure 1), *C. adoensis *accessions from East Africa cluster with the East African *C. grandiflora *and *C. schliebenii*, while pubescent *C. adoensis *accessions 1 and 6 from South Africa (originally described as *C. pubescens*) cluster with a glabrous *C. adoensis *9 from southern Zambia. In the nuclear *LFY *tree (Figure 2), *C. adoensis *2, which in the plastid tree clustered with East African plants, instead groups with *C. adoensis *9 (South African *C. adoensis *plants did not yield *LFY *sequences). The ITS alignment (Figure 3) reveals that single individuals of *C. adoensis *can have two kinds of sequences: *C. adoensis *5 from Zambia (sister to *adoensis *2 in the plastid DNA data; Figure 1) harbors sequences matching *C. adoensis *1 from South Africa as well as sequences matching *C. adoensis *2 from Namibia. Second, in the plastid tree, East African *C. adoensis *are distant from *C. aurantiaca*, while in the nuclear tree they are in a polytomy with *C. aurantiaca *and *C. adoensis *9.

## Discussion

At the outset of this study, we expected minimally two biome shifts, this being the number required to explain the presence of *Coccinia *in semi-arid habitats, woodland, and forest. Instead, we found six statistically significant biome shifts among close relatives (marked in Figure 4b). However, this is still fewer than if the habitats were distributed on the phylogeny at random. The onset of *Coccinia *diversification dates to just 6.9 Ma ago, a time when the warm and humid climate began to become cooler and drier. Climatic conditions then continued to oscillate during the Pliocene and Quaternary (*Background*). Additionally, the East African rifting led to aridification and more open grasslands starting at 7 - 8 Ma ago [15], [19]. Depending on species' ecological tolerances, these climate fluctuations must have caused range reduction and fragmentation, or expansion and merging. The likely ancestral precipitation preferences of the *Coccinia *clade remain unresolved (Figure 4b); the sister genus, *Diplocyclos*, which comprises four species, is restricted to rain forest and semi-deciduous woodlands [31].

The 12 forest species of *Coccinia *all have discontinuous distributional ranges, as exemplified by *C. grandiflora *(Figure 7), fitting with forest expansion during Pleistocene interglacials that likely reconnected most forest refugia [33]. Survival in persisting refugia probably explains the populations of *C. subsessiliflora *in the southern Sudanese Imatong Mts. (Figure 5a), of *C. barteri *in the mountain region between Zimbabwe and Mozambique, and of *C. mildbraedii *in the Eastern Arc Mts. For *C. heterophylla*, which occurs in the Angola Escarpment at 15°30'S (Figure 5a), mist-saturated local vegetation pockets [34] may have offered survival possibilities during dry periods, while the presence of *C. mildbraedii *in the Kenyan highlands (Figure 5a), may result from introduction by humans (fide label information on the specimen *J. B. Gillett *20185, MO, NHT). It nevertheless shows that Central African species would probably find suitable habitats in East Africa if forest expansion advanced further.

The *Coccinia barteri *clade is interesting in containing two rain forest species (*C. longicarpa *and *C. keayana*) with overlapping distributions (Figure 5a) and co-occurrence in the same habitats (e.g., in the Banco Forest Reserve, Abidjan, Ivory Coast). They likely descend from a widespread ancestral species, the range of which became fragmented during the cool/dry mid-Pliocene, with *C. longicarpa *becoming restricted to southwestern Ghana and *C. keayana *to Liberia, fitting with Maley's [25] refugia. Today, *C. longicarpa *is also distributed east of the Dahomey Gap (arrow in Figure 5), an abrupt climatically induced rain forest disjunction in West Africa. Although forest fragmentation during glacial periods likely was severe, present range disruptions in Central and West Africa seem to date only to the recent Holocene [35]. Recurrent fragmentation and reconnection of populations during the Pleistocene apparently led to hybridization and introgression, which would explain the high morphological and genetic variability in *C. barteri *(Figure 1, Table 3).

Among the few species of *Coccinia *that appear to have originated during and since the Pleistocene are the forest species *C. grandiflora/C. schliebenii *from East Africa and *C. quinqueloba/C. mackenii *(the latter identical in the markers sequenced here) from South Africa. Each pair comprises morphologically similar species with partly overlapping ranges (Figure 5 and 7). The stronger aridity in East and southern Africa compared to Central and West Africa seems to have led to Pleistocene allopatric speciation in these aridity intolerant species. That the range of *C. schliebenii *extends into Ethiopia and the Didinga Mts. in southeastern Sudan (Figure 7), which have similar amounts of precipitation, probably reflects long-distance seed dispersal by birds [36,37], rather than remnant populations from a once continuous range. This is because intervening forests, such as those of the Usambara Mts. and Mt. Kenya, have been well collected, yet have not yielded *C. schliebenii*.

*Coccinia *species of the *rehmannii *clade and other dry-adapted species occur on either side of the Miombo belt (with 3 - 6 months of aridity), but are absent from the belt itself (Figure 7). The reason does not appear to be the belt's poor lateritic soils [38] since *Coccinia *species can grow on such soils (*C. microphylla*: *R. Wingfield *1351 and 2893, DSM; *C. trilobata*: *R. Polhill *&*S. Paulo *962, K), and so are *C. grandis *(*E. Westphal *&*J. M. C. Westphal-Stevels *1385, MO, WAG; *J. J. Lavranos *&*S. Carter *23258, MO) and *C. sessilifolia *(*G. Germishuizen *9384, MO; *S. E. Chadwick *280, MO). Fire is an unlikely explanation too, since *Coccinia *species have tubers and can re-sprout. During the Pleistocene, the Miombo belt apparently was crossable for ostriches and antelopes [39,40], making its barrier role for *Coccinia *even more difficult to understand.

## Conclusions

The at least six biome shifts among the 27 species of *Coccinia *analyzed here may be an underestimate because of our assignments of species into just three broad biome types, semi-arid habitats, woodland, and forest. A fuller understanding of the physiological traits behind tolerated precipitation regimes in *Coccinia *would require transplants or common garden experiments [41]. The present results, based on occurrence data and ecological information from herbarium specimen labels, however show that changes in ecological tolerances (especially drought tolerance) have played an important role in the diversification of *Coccinia*. A strength of this study is that it is based on consistent species concepts and geo-referenced data for well over 1000 collections.

## Methods

### Species Distribution Analysis and Biome Coding for Ancestral State Reconstruction

The first author surveyed c. 1400 specimens from 25 herbaria (B, BM, BR, COI, DSM, E, EA, FT, GAT, GOET, H, HBG, HEID, K, M, MO, MSB, NHT, P, S, UBT, WAG, and partly C, LISC, and LISU). Collecting localities (and some ecological information) were taken from herbarium specimen labels and geo-referenced 1189 of them with Google Earth, Google Maps (Google Inc., Mountain View, CA, USA), and online maps of the Perry-Castañeda Library Map Collection (http://www.lib.utexas.edu/maps/). Climate data were extracted from the WorldClim database (http://www.worldclim.org; [42]) using DIVA-GIS 7.1.6.2 (http://www.diva-gis.org). The number of arid months was calculated by counting how often the arithmetic mean of the monthly minimum and maximum temperature [°C] is larger than half of the monthly precipitation [mm] [43]. For ancestral state reconstruction, we assigned 24 species to one of three habitat categories: Semi-arid habitats (which includes semi-desert, bushlands, semi-arid savannas), woodland (including habitats such as mountain shrublands, humid grasslands, semi-humid savannas), or forest (including semi-deciduous forest, lowland rain forest, and mountain forest). Assignment of specimens/species to habitats followed information given on herbarium specimen labels and the WorldClim data for the respective location. *Coccinia grandis*, which occurs in African bushlands and savannas as well as in ruderal sites throughout the humid tropics, was coded as "semi-arid habitats" to reflect its drought tolerance.

Differences in annual precipitation and number of arid months tolerated by members of a species pair or a small clade were tested by pair-wise Mann-Whitney U tests in SPSS 13.0 (SPSS, Chicago, IL, USA). Trait reconstructions were carried out in Mesquite 2.71 [44] under maximum likelihood optimization, using the maximum clade credibility tree (with median heights) from the plastid DNA data obtained from BEAST (below) and Lewis' [45] Markov k-state one parameter model.

Finally, we tested whether the number of pairs of species in which members share the same niche preferences differs from that obtained if species habitat distributions were distributed on the tree at random.

### Molecular Phylogenetic Taxon Sampling and Methods

We sampled 25 of the 27 species of *Coccinia *for several plastid and/or nuclear DNA markers. Only the poorly collected species *Coccinia pwaniensis *Holstein [46] and *C. variifolia *A. Meeuse could not be included. Trees were rooted on four outgroups, *Cucumis sativus, Cucumis hirsutus, Peponium vogelii*, and *Scopellaria marginata*, based on studies that included species from all African genera of Cucurbitaceae [47,48]. DNA was extracted from 3 - 20 mg of leaf tissue from herbarium specimens or silica-dried plant material, using Macherey-Nagel plant extraction kits (Macherey-Nagel, Düren, Germany). For some samples, the lysis buffer was altered by adding sodium meta bisulfite (S9000, Sigma-Aldrich Chemie GmbH, Munich, Germany) to a 10 mM final concentration [49]. PCR reactions used standard conditions, except for the addition of bovine serum albumine (Fermentas, St. Leon-Rot, Germany). We amplified the plastid intergenic spacers *trn*S^GCU^-*trn*G^UCC ^and the *rpl*20-*rps*12 using the primers of Hamilton et al. [50], the *ndh*F-*rpl*32 spacer using the primers of Shaw et al. [51], the *mat*K gene and *trn*K intron using the primers of Yokoyama et al. [52], and the *trn*L^UAA ^intron and *trn*L^UAA^-*trn*F^GAA ^spacer using the universal primers of Taberlet et al. [53]. PCR products were checked on a 1% agarose gel, and those with multiple bands were run on a 2% agarose gel, cut, and treated with the Wizard SV PCR clean-up kit (Promega GmbH, Mannheim, Germany), following the manufacturer's instructions. Phusion high fidelity DNA Polymerase (Finnzymes, Espoo, Finland) was used for recalcitrant and low-concentrated samples and to amplify the 2^nd ^intron in the nuclear *LFY *gene. Primers for this region came from Volz and Renner [54] and from a M.Sc. thesis carried out in our lab [55]: LFYubiq F1: 5'-CAY CCN TTY ATH GTN CAN GAR CC-3'; LFYubiq-R1: 5'-GCR TAR CAR TGN ACR TAR TGN CKC AT- 3'.

The complete ITS region was amplified using the primers of Balthazar et al. [56]. Where necessary, we used cloning to assess within-plant sequence divergence, focusing on the polymorphic species. For cloning, we ligated PCR products into plasmids of the Promega pGEM-T Vector system (Promega). Plasmids were transformed in ultra competent *E. coli *DH5alpha strains [57]. Positive (white) plasmid colonies were picked from the ampicillin blue/white selection agar plates, solved in 4 ml LB medium with 100 mg/ml ampicillin, and grown over-night at 37°C. Plasmids were obtained using GeneElute Miniprep Kit (Sigma-Aldrich) and directly amplified with primer oligonucleotides and settings as mentioned above. PCR products were purified and sequenced, using the same primers. Sequencing was performed on an ABI Prism 3130 Avant capillary sequencer using Pop-7 polymer (Applied Biosystems, Foster City, CA, USA), and sequences were edited with Sequencher v. 4.6 (Gene Codes, Ann Arbor, MI, USA).

### Alignment, Phylogenetic Inference and Divergence Time Estimation

Sequences were aligned by eye, using MacClade v. 4.06 [58]. We excluded ambiguously alignable regions and structurally homoplastic sections. This concerned a total of 219 alignment positions in the plastid data (mostly microsatellites) and 42 nucleotides in the nuclear *LFY *matrix. Tree inference relied on maximum likelihood and was carried out in RAxML v. 7.2.2 [59], with the final parameter evaluation done under the GTR + Γ substitution model. We used this model to approximate the best-fit models found with Modeltest v. 3.7 [60], which under a hierarchical likelihood ratio test indicated the F81 + I + Γ model as the best fit for the combined plastid data, while under the Akaike information criterion it found TVM + I + Γ as the best fit. Statistical support for individual nodes was assessed via bootstrapping with 100 replicates [61]. Nucleotide insertions and deletions (indels) were plotted on the resulting tree to test whether they contained phylogenetic information.

Divergence times were inferred using the program BEAST v. 1.5.2 [62], which employs a Bayesian Markov chain Monte Carlo (MCMC) approach to co-estimate topology, substitution rates, and node ages. The input data consisted of a matrix comprising 26 accessions from 24 species of *Coccinia *(*C. mackenii *and *C. quinqueloba *had identical sequences, and the former was therefore removed) and six outgroup accessions. There are no *Coccinia *fossils, and we therefore used a secondary calibration from a fossil-calibrated Cucurbitaceae-wide dating analysis [48] that obtained an age of 15 million years (Ma), with a standard deviation of 3.0 Ma, for the split between *Coccinia *and *Diplocyclos*. We used this age as a prior constraint on the root node of *Coccinia*, with a normal distribution and a standard deviation (SD) of 2.6 Ma. A SD of 2.7 Ma (or larger) resulted in poor convergence of the MCMC chain. We used a strict clock model (which we preferred because we have a single secondary constraint), a Yule process tree prior, and MCMC chains of 10 million generation length, with parameters sampled every 1000^th ^generation. The first 20% of the trees was discarded as burn-in, and convergence and mixing of the chain were assessed by consistency across runs, inspection of trace plots in the program TRACER v. 1.4.1 [62], and from the effective sample sizes (ESS), which were well above 1000 for all estimated parameters. Four independent BEAST runs yielded the same maximum clade credibility topology, and we also ran an analysis without the data to verify that the effective priors do not contradict the original priors and to assess the informativeness of the data. The cut-off for nodes to be considered in the chronogram was ≥ 0.98 posterior probability.

## Authors' contributions

NH generated sequences and alignments, distribution data, performed data analyses, and worked on the manuscript. SSR conceived the study and drafted the manuscript. Both authors read and approved the final manuscript.
